# *MYC* amplification is common in cancer of the stomach and gastroesophageal junction, correlates with male sex and reduced response to neoadjuvant therapy

**DOI:** 10.1186/s12885-026-16074-3

**Published:** 2026-04-24

**Authors:** Ole Biegler, Hans-Michael Behrens, Jochen Haag, Thomas Becker, Steffen Markus Heckl, Silke Lüschen, Christoph Röcken

**Affiliations:** 1 Department of Pathology, Christian-Albrechts-University, University Hospital Schleswig-Holstein, Arnold-Heller-Str. 3, House U33, Kiel, D-24105 Germany; 2 Department of General, Visceral, Thoracic, Transplant and Pediatric Surgery, Christian-Albrechts-University, University Hospital Schleswig-Holstein, Kiel, Germany; 3 Department of Internal Medicine II, Christian-Albrechts-University, University Hospital Schleswig-Holstein, Kiel, Germany

**Keywords:** Gastric cancer, MYC, *MYC* amplification, Sexual dimorphism, Neoadjuvant chemotherapy

## Abstract

**Background:**

We aimed to demonstrate that *MYC* amplification in adenocarcinomas of the stomach (GC) and gastroesophageal junction (GEJ) is of tumor biological significance and exhibits intratumoral heterogeneity.

**Methods:**

*MYC* amplification was analyzed by fluorescence in situ hybridization using whole mount tissue sections obtained from resection specimens of 460 chemotherapy naive (surgery only) and 122 neoadjuvantly treated GC-GEJs. The extent of intratumoral heterogeneity was quantified and a MYC score was established.

**Results:**

The number of *MYC*-amplified tumor cells varied largely ranging from few scattered single cells (< 20 per high power field) up to all tumor cells. MYC score 1 was found in 276 cases (47.4%). Split by cohorts, 199 (43.3%) tumors of the chemotherapy naïve (surgery only) and 77 (63.1%) tumors of the neoadjuvant cohort were sorted into the category MYC Score 1. These tumors were frequently located in the gastroesophageal junction, showed non-diffuse differentiation according to Lauren, and favored male sex. Additionally, correlations with higher T- and N category were observed. Overall survival and tumor-specific survival correlated negatively with the MYC score.

**Conclusions:**

*MYC* amplification is common in GC-GEJ, most often heterogeneously distributed, and associated with reduced therapy response. Most interestingly, *MYC* amplification is far more common in men, providing evidence for sex-specific disease mechanisms, and in tumors that show little response to neoadjuvant chemotherapy, suggesting a link to therapy resistance.

**Supplementary Information:**

The online version contains supplementary material available at 10.1186/s12885-026-16074-3.

## Background

Gastric cancer (GC) is one of the most common cancers worldwide. With over a million new cases in 2020, it ranks fifth in incidence [1]. The rates among men are twofold higher than those observed in women [1]. Even though incidences are generally decreasing, the demographic change and increases in incidences in people aged < 50 years could become future challenges [2]. Common risk factors are infection with *Helicobacter pylori* (H. pylori) or Epstein-Barr virus (EBV). Certain lifestyle factors like tobacco and alcohol consumption, a diet with high salt intake, obesity, familial predisposition and older age further increase GC risk [3–5]. Since GC remains asymptomatic for a long time, patients are often diagnosed at an advanced stage limiting therapeutic options. The standard regimen with curative intent in locally advanced GC is perioperative FLOT (fluorouracil, leucovorin, oxaliplatin and docetaxel) [6]. Targeted therapies are currently being explored in this setting, while they are standard of care in the palliative setting, targeting molecules like programmed cell death protein 1 (PD-1) or the human epidermal growth factor receptor 2 (HER2) [7]. A major obstacle targeted therapies face is intratumoral heterogeneity [8]. This phenomenon is well known to occur in GC and leads to challenges during molecular characterization and therapy [9, 10]. Previously, using multiregional sequencing, we noticed intratumoral heterogeneity of *MYC* amplification in GC [11]. C-MYC is part of the MYC oncoprotein family which includes C-MYC, N-MYC and L-MYC. Located on chromosome 8q24, C-MYC acts as a transcription factor and affects numerous target genes [12, 13]. MYC promotes various hallmarks of cancer such as proliferation, programmed cell death, angiogenesis and differentiation for example [14, 15]. Additionally, *MYC* was shown to be frequently amplified in tumor samples across 33 cancer types of *The Cancer Genome Atlas* and proven to be mutually exclusive with important oncogenic drivers like *PTEN*, *BRAF*, *APC* and *PIK3CA* at the pan-cancer level [16]. *MYC* amplification has been found in many cancer entities like small cell lung cancer [17], breast cancer [18] and colon cancer [19]. In relation to GC, five studies correlated *MYC* amplification to clinicopathological patient characteristics with limited patient numbers [20–24]. Currently, no data are available on large cohorts of Caucasian patients analyzing the extent of *MYC* amplification in GC and assessing the extent of intratumoral heterogeneity. Additionally, there is a lack of data on *MYC* towards patients who underwent neoadjuvant treatment protocols and adenocarcinomas of the gastroesophageal junction (GEJ). Burbano et al. studied a single case of GEJ [20], Khaleghian et al. 12 [23] and de Souza 52 [22]. To fill this gap of information, we performed a comprehensive study on a large and well characterized cohort of Caucasian patients with adenocarcinomas of the stomach and gastroesophageal junction (GC-GEJ). We aimed to shed light on the differences between *MYC* deregulation in chemotherapy naive and neoadjuvantly treated GC-GEJs and demonstrate that the amplification of *MYC* is of tumor biological significance in both clinical settings.

## Materials and methods

### Patients and tumor samples

We identified all patients from the archive of the Department of Pathology, University Hospital Schleswig-Holstein, Campus Kiel, who had undergone partial or total gastrectomy for adenocarcinoma of the gastroesophageal junction or stomach between the years of 1997 and 2022. Adenocarcinomas of the gastroesophageal junction and stomach were grouped together in this study, as *The Cancer Genome Atlas Research Network* analyses have shown that both share the same molecular subtypes [25, 26]. Patients were included in this study if histological examination confirmed the diagnosis of adenocarcinoma. Furthermore, the availability of survival data was required for inclusion. Exclusion criteria were any other type of tumor, missing clinicopathological patient characteristics, missing consent from the patient and if the tumor was not the primary tumor. Additional exclusion criteria for the neoadjuvant cohort were any other therapy scheme than FLOT, a missing neoadjuvant therapy and cases that showed complete remission under neoadjuvant treatment. Necessary patient characteristics were retrieved from the database including sex, age at the time of surgery, tumor localization, histological subtype according to Lauren, size of the primary tumor (pT category), differentiation of the tumor, type of surgery, depth of tumor invasion, number of resected lymph nodes, number of lymph nodes with metastases (pN category), lymph node ratio, presence of distant metastases (pM category), tumor stage according to UICC, invasion of lymph vessels (pL category), invasion of blood vessels (pV category), perineural invasion (pPn), residual tumor status (pR category) and grading of the tumor regression (TRG) according to Becker et al. [27]. With regard to tumor regression published by Becker et al. [27], the entire former tumor bed was embedded in paraffin and centrally assessed by two independent board-certified surgical pathologists, in order to account for inter-observer variability. The evaluation was centralized at the Department of Pathology at the University Hospital of Schleswig-Holstein in Kiel, Germany. The results of each assessment were obtained from the patients’ histopathology reports. A Becker regression score of TRG1a (complete remission), TRG1b, TRG2 or TRG3 were assigned, if no residual tumor/tumor bed, < 10% residual tumor/tumor bed, 10–50% residual tumor/tumor bed or > 50% residual tumor/tumor bed were seen, respectively [27]. The date of patient death was obtained from the Epidemiological Cancer Registry of the state of Schleswig-Holstein, Germany. Follow-up data from patients still alive were obtained from hospital records and general practitioners. All patients turned out to be Caucasians.

### Study design

592 GC samples met the study criteria and were divided into two different patient cohorts: (1) The chemotherapy naive cohort included 470 patients and was treated by surgery only without neoadjuvant therapy. (2) The neoadjuvant cohort included 122 patients, underwent neoadjuvant treatment according to the FLOT protocol. The clinical and outcome data of the neoadjuvant cohort had been described in detail previously [28]. Whole mount tissue sections (WMTS) were cut from the primary tumors of both cohorts and forwarded to fluorescence in situ hybridization (FISH). Tumors that showed no signals or invalid signals were then excluded from further analyses.

### Histology

All specimens were obtained during routine (esophago-)gastric surgery from oncological treatment of GC-GEJ. Grossing and primary surgical pathological assessment had been performed by board certified surgical pathologists. Tissue specimens had been fixed in formalin and embedded in paraffin. Deparaffinized WMTS were stained with hematoxylin and eosin. Histological re-examination of primary tissue sections was carried out for all cases to assure all inclusion criteria were met. Tumors were classified according to Lauren and re-examined by two surgical pathologists [29]. pTNM-stage of all study patients was determined according to the 8th edition of the UICC guidelines [30].

### Assessment of clinicopathological characteristics

The following clinicopathological patient characteristics were obtained from previous studies that were performed at the Department of Pathology, University Hospital Schleswig Holstein, Kiel, Germany. Infection with *H. pylori* was evaluated by modified Giemsa staining and polymerase chain reaction [31]. Epstein-Barr-Virus infection was detected with the EBER-probe and the BondMax-detection system (Leica Biosystems, Illinois, USA) [32]. The Her2/neu status was analyzed by immunohistochemistry and in situ hybridization [33]. The expression and amplification of *MET* was analyzed by immunohistochemistry and chromogenic in situ hybridization [34]. The expression of p53 was analyzed by immunohistochemistry and assessed using the Histoscore (HScore) [35]. The HScore was calculated according to the formula: HScore=[0×percentage of immunonegative tumor cells]+[1×percentage of weakly stained tumor cells]+[2×percentage of moderately stained tumor cells]+[3×percentage of strongly stained tumor cells], resulting in a possible HScore between 0 and 300. Tumor cells without detectable staining were scored as 0. The maximum possible HScore was 300, if all cells of a given tumor sample showed strong staining: [0 × 0%]+[1 × 0%]+[2 × 0%]+[3 × 100%] = 300. The cohort was then split at the median, into “low” (HScore < 92) and “high” (HScore ≥ 92) groups, with the “high” group approximating cases with mutated *TP53* as described in detail previously [35]. Microsatellite-instability (MSI) was analyzed as previously described [36]. The assessment of “Stroma AReactive Invasion Front Areas” (SARIFA) was carried out as the criteria of the original authors suggest [37]. Tumor budding was analyzed according to the suggestions of the International Tumor Budding Consensus Conference (ITBCC) [38].

### Fluorescence in situ hybridization

Whole mount tissue sections were used for FISH analysis. The tissue sections were dewaxed and rehydrated in a descending alcohol series. They were then washed in deionized water (dH_2_O) and incubated for 2 × 10 min in 1 N citrate buffer and washed again in dH_2_O. Proteolysis was initiated by placing the tissue sections in 0.1 N HCl with 0.01% Proteinase K for 10 min (Cat. # EO0491, Thermo Fisher Scientific, Waltham, Massachusetts, USA). After washing with dH_2_O, dehydration in an ascending alcohol series and air drying, the sections were fully covered with the probe solution (SPEC MYC/CEN8 Dual Color Probe, Cat. # Z-2092-200, ZytoVision GmbH, Bremerhaven, Germany) and sealed. Overnight, the slides were placed in a ThermoBrite (Abbott Molecular, Chicago, Illinois, USA) and hybridized at 95 °C for 10 min followed by 37 °C for the remaining time. On the following day, the slides were washed with a Standard Saline Citrate (SSC) buffer and stained using Vectashield Antifade Mounting Media with 4´,6-diamidino-2-phenilindole (DAPI) (Cat. # VEC-H-1200, Biozol, Eching, Germany).

### Assessment of fluorescence in situ hybridization

**Fig. 1:**
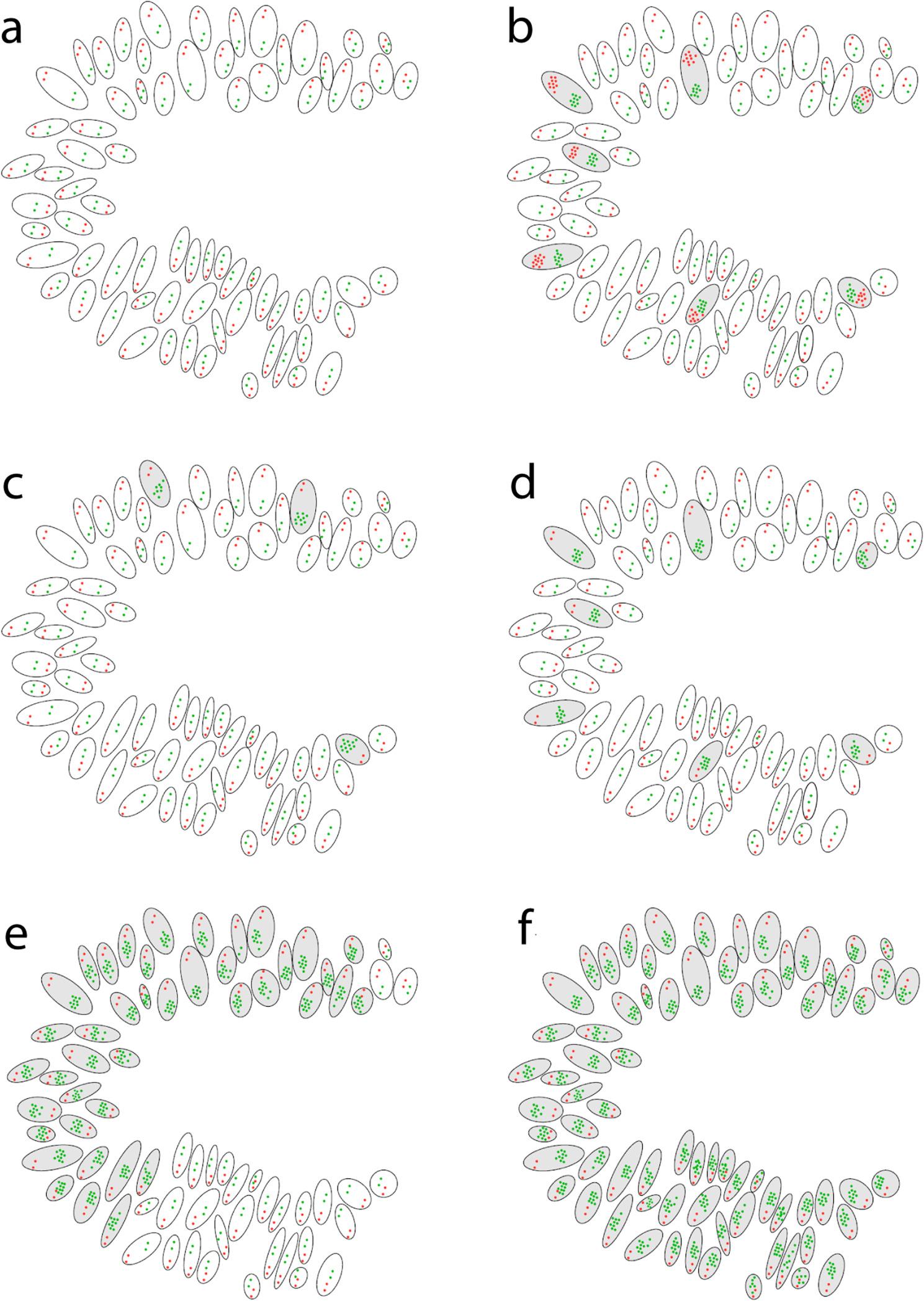
Graphical illustration of assessed patterns of *MYC* amplification by fluorescence in situ hybridization. Illustration showing tumor tissue with: (**a**) no amplification; (**b**) cells with gene count ≥ 6.0 in ≥ 20 amplified single cells per 10 high power fields; (**c**) < 20 amplified single cells per 10 high power fields; (**d**) ≥ 20 amplified single cells per 10 high power fields; (**e**) heterogeneous amplification in 25-50% of the tumor cells; (**f**) homogeneous amplification in all tumor cells (100%). Green signals = MYC, red signals = CEN8

The results of the FISH were evaluated using a fluorescence microscope Nikon Eclipse Ni (Nikon Corporation, Minato, Japan). The WMTS were evaluated to avoid missing any tumor tissue and to ensure that possible heterogeneity within the tumor was detected. Thirty representative coherent tumor cells were counted and the number of green (*MYC*) signals was divided by the number of red (*CEN8*) signals. If the *MYC*/*CEN8* ratio was ≥ 2 the tumor was classified as amplified. A ratio below 2 classified a tumor as not amplified. A ratio between 1.8 and < 2 led to an additional count of 30 tumor cells. Furthermore, the presence of *MYC*-clusters was noted. Considering a possible gene-dose effect, additionally, the gene count was calculated by dividing the number of counted *MYC* signals by the number of counted cells. A gene count ≥ 6.0 was also defined as *MYC*-amplified. Tumors that showed heterogeneity within the tumor tissue were estimated regarding the proportion of *MYC-*amplified tumor cells. Four subgroups were used to categorize heterogeneity: less than 25% of the tumor cells, 25–50%, 51–75% and more than 75% of the tumor cells. In contrast to other common counting procedures in FISH/CISH analysis where only coherent cells were assessed for amplification, we recognized in many samples a high number of single cells with *MYC* amplification distributed throughout the tumor tissue. Single cells with a ratio ≥ 2.0 and/or a gene count ≥ 6.0, but not within the presence of 30 coherent amplified cells, were defined as “amplified single cells”. Additionally, if a tumor showed isolated amplified single tumor cells, the amount of amplified single cells was counted using 10 high power fields (microscopic fields at x100 magnification), which are equivalent to 0.36 mm^2^. Figure 1 illustrates and summarizes the assessment of FISH.

### Statistical analysis

For the statistical analysis SPSS version 29.00.02.00 (IBM Corporation, Armonk, New Jersey, USA) was used. We used overall survival and tumor specific survival as endpoints for Kaplan-Meier analysis, respectively. Overall survival was defined as the time from surgery until either death from any reason or last visit. Tumor specific survival was defined as the period starting from the time of surgery until death from GC-GEJ or last visit. Significance of differences between survival curves was tested with the log-rank test. The Fisher´s exact test was used for correlations between nominal variables. Correlations between ordinal variables were tested with the help of the Kendall´s tau test. To account for the false discovery rate, the Benjamini-Hochberg (Simes) method was applied to the pool of all p-values of this study [39]. We highlighted the p-values that lost significance. The Cutoff Finder web application was used to determine the optimal dichotomization of number/percentage of MYC amplified tumor cells into MYC score 0 and 1 groups, targeting patient survival as a surrogate marker for biological effects [40]. Values of *p* ≤ 0.05 were classified as significant. To test the independence of specific variables we used binary logistic regression. Multivariable survival analysis was carried out using Cox regression separately for both cohorts. All variables that showed a p-value less than 0.1 in their respective univariable survival analysis were included into our initial Cox model, i.e., Lauren classification, pT category, pN category, pM category, lymph node ratio, pL category, pV category, pPn category, pR status, MSI status, MET status, SARIFA, tumor budding (Bd), and MYC score. The backward-LR method in SPSS iteratively removed all variables from the model that suffered a drop of their respective p-value below 0.05.

## Results

All of the inclusion criteria were met by 592 patients, who were then sent to FISH. During microscopic examination, ten cases were excluded due to invalid signals, leaving 582 assessable cases: 460 in the chemotherapy naive cohort and 122 in the neoadjuvant cohort. Table 1 summarizes patient demographics and the clinicopathological patient characteristics.

**Table 1:**
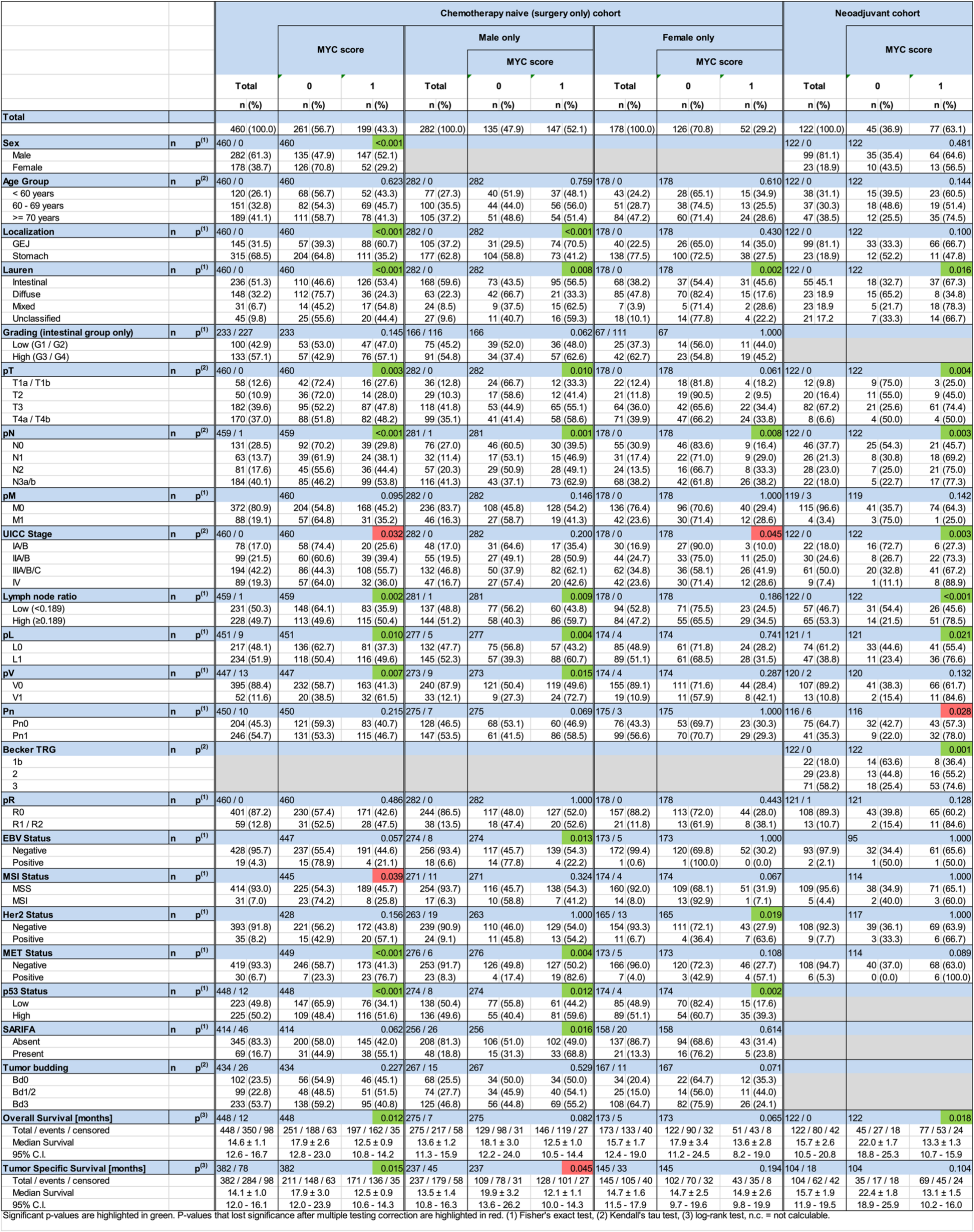
Correlation of MYC score with clinicopathological patient characteristics

### *MYC* amplification in gastric cancer

**Fig. 2:**
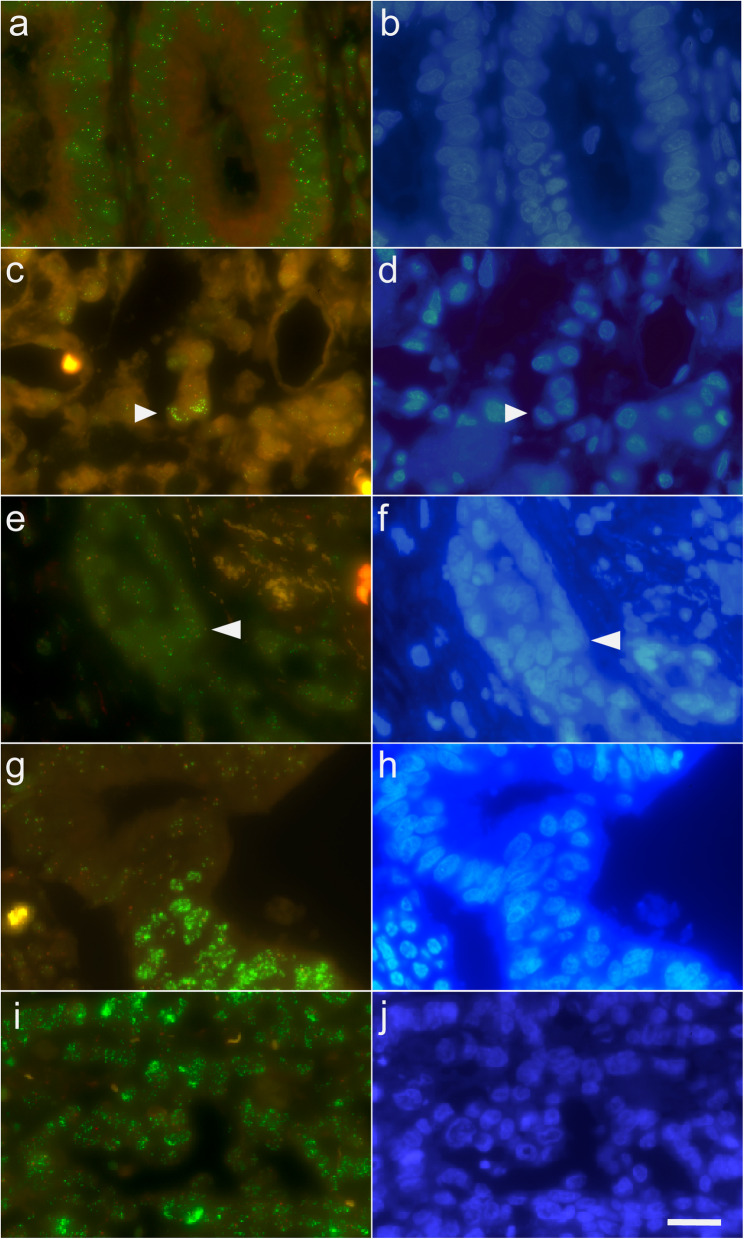
Fluorescence in situ hybridization with *MYC/CEN8* probe. MYC/CEN8 signals are shown on the left (**a**, **c**, **e**, **g**, **i**) and the corresponding DAPI stainings are shown on the right (**b**, **d**, **f**, **h**,** j**). (**a**, **b**) no MYC amplification; (**c**, **d**) two amplified single cells (arrow heads); (**e**, **f**) cells with a gene count ≥ 6.0 (arrow heads); (**g**, **h**) heterogeneous amplification; (**i**, **j**) homogeneous amplification. Original magnification: 400-fold. Green signals = 50 µm

Among the entire cohort of 582 assessable cases, *MYC*-amplified tumor cells, defined by *MYC*/*CEN8* ratio ≥ 2.0 and/or gene count ≥ 6.0, were found in 332 (57.0%) cases (244 chemotherapy naive cohort, 88 neoadjuvant cohort). Gene clusters were noted in 208 (35.7%) specimens. The number of *MYC*-amplified tumor cells varied largely ranging from few scattered single cells (< 20 per high power field) up to all tumor cells. The degree of amplification was highly variable between patient samples and within individual tumors, even in homogeneous tumors in which all tumor cells were *MYC*-amplified. Thus, *MYC* amplification in GC-GEJ shows a highly heterogeneous pattern (Fig. 2). In order to facilitate statistical analyses, we then categorized the amounts of amplified tumor cells into eight groups, i.e. < 20 *MYC*-amplified single tumor cells per ten high power fields (with a *MYC/CEN8* ratio ≥ 2.0 and/or a *MYC* gene count ≥ 6.0), ≥ 20 *MYC*-amplified single tumor cells per ten high power fields (with a *MYC/CEN8* ratio ≥ 2.0 and/or a *MYC* gene count ≥ 6.0), coherent groups of amplified tumor cells covering < 25% of the tumor area, 25–50%, 51–75%, 76–99% and 100% with either *MYC*/*CEN8* ratio ≥ 2.0 and/or gene count ≥ 6.0 (Table 2).

**Table 2:** Evaluation of MYC amplification by fluorescence in situ hybridization in whole mount tissue sections from 592 patients. Comparison of surgery only and neoadjuvantly treated cohort

	Surgery only cohort	Neoadjuvant cohort	Total
	[*n* (%)]	[*n* (%)]	[*n* (%)]
Number of cases examined	470	122	592
Number of cases excluded	10	0	10
**Number of cases included in the statistical analyses**	**460**	**122**	**582**
MYC negative	**216 **(47.0)	34 (27.9)	250 (43.0)
< 20 amplified single cells per 10 high power fields	**45 **(9.8)	11 (9.0)	56 (9.6)
≥ 20 amplified single cells per 10 high power fields	**67 **(14.6)	35 (28.7)	102 (17.5)
< 25% tumor area amplified	**34 **(7.4)	18 (14.8)	52 (8.9)
25–50% tumor area amplified	**21 **(4.6)	5 (4.1)	26 (4.5)
51–75% tumor area amplified	**13 **(2.8)	5 (4.1)	18 (3.1)
76% -99% tumor area amplified	**12 **(2.6)	5 (4.1)	17 (2.9)
100% tumor area (= homogenous) amplified	**52 **(11.3)	9 (7.4)	61 (10.5)
MYC score 0	**261 **(56.7)	45 (36.9)	306 (52.6)
MYC score 1	**199 **(43.3)	77 (63.1)	276 (47.4)

Dichotomization of patient cohorts at median values may not reflect biological relevant cutoffs, as it was previously shown by our group, e.g., for neutrophil counts in GC [41]. Therefore, we explored an alternative dichotomization approach for MYC score using Cutoff Finder [40] and patient survival as outcome measure. Cutoff Finder is a web-based application, which enables detection of optimal biomarker cutoffs. Using the chemotherapy naive cohort, the prognostically relevant cutoff was set as follows: ≥ 20 amplified single cells in 10 high power fields (i.e., *MYC/CEN8* ratio ≥ 2.0 and/or a *MYC* gene count ≥ 6.0; Table 1). Thus, tumors that showed no *MYC* amplification or < 20 *MYC*-amplified single tumor cells per ten high power fields were categorized as MYC score 0. Tumors with ≥ 20 amplified single tumor cells per ten high power fields, and all tumors with coherent tumor cell areas (with a *MYC/CEN8* ratio ≥ 2.0 and/or a *MYC* gene count ≥ 6.0) were categorized as MYC score 1. Following this dichotomization, 199 (43.3%) GC-GEJs of the chemotherapy naive and 77 (63.1%) GC-GEJs of the neoadjuvant cohort were categorized as MYC score 1 (Table 1).

### Correlation of MYC score with clinicopathological patient characteristics

We than aimed to assess the tumor biological significance of *MYC* amplification and correlated the MYC score with various clinicopathological patient characteristics separately for the chemotherapy naive and neoadjuvant cohort. In the chemotherapy naive cohort, the MYC score correlated significantly with sex, tumor localization, tumor type according to Lauren, pT category, pN category, lymph node ratio, lymph and blood vessel invasion (Table 1). In addition, a significantly positive correlation was found with MET- and p53 status (Table 1). Due to the highly significant difference between men and women (52.1% vs. 29.2% MYC score 1) we analyzed men and women of the chemotherapy naive cohort separately. This showed that tumor localization, tumor growth (pT category), lymph node ratio, lymph vessel invasion, blood vessel invasion, MET status and SARIFA correlated only in men positively with the MYC score. In addition, a negative correlation was found with EBV (Table 1). Binary logistic regression showed that the sex specific difference was not dependent on tumor type according to Lauren or tumor localization. In women of the chemotherapy naive cohort, an additional positive correlation was found with HER2 status (Table 1). In the neoadjuvant cohort, no significant difference was found between men and women. However, MYC score correlated significantly with tumor type according to Lauren, pT category, pN category, UICC stage, lymph node ratio, lymph vessel invasion and MET status. Additionally, the number of cases with MYC score 1 steadily increased with decreasing therapeutic response as assessed by the tumor regression score according to Becker. Next, the correlation between the MYC score and clinicopathological patient characteristics was examined separately for GEJ and GC. Again, after correction for multiple testing, a significant association was found with gender (GEJs), tumor type (GCs), nodal status (GCs), lymph node ratio (GCs) and MET status (GCs) in chemotherapy-naive cases (Supplementary Table 1), and with local tumor growth (pT category; GEJs), UICC stage (GEJs), the lymph node ratio (GEJs), pPn category (GEJs), and the degree of tumor regression (GEJs) in neoadjuvantly treated tumors (Supplementary Table 1).

**Fig. 3:**
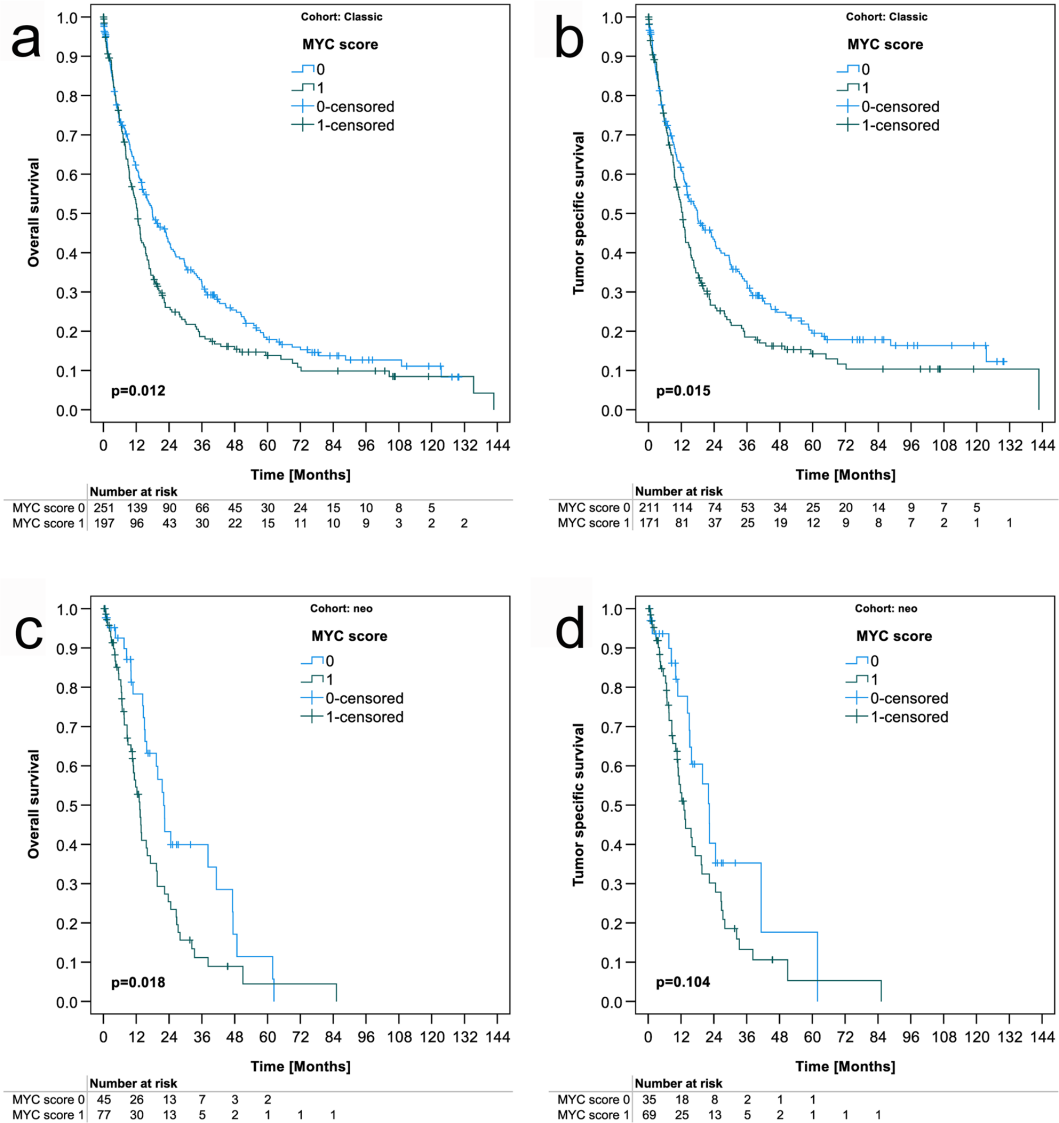
*MYC* amplification dependent survival analysis by Kaplan Meier Plots. Comparison of MYC Score 0 and MYC Score 1 in the chemotherapy naive cohort: (**a**) overall survival; (**b**) tumor specific survival. Comparison of MYC Score 0 and MYC Score 1 in the neoadjuvant cohort treated with FLOT regimen: (**c**) overall survival; (**d**) tumor specific survival

Finally, we correlated the MYC score with patient survival. In the chemotherapy naive cohort, the median survival of MYC score 1 was significantly shorter compared with MYC score 0 (median overall survival time 12.5 ± 0.9 months vs.17.9 ± 2.6 (Fig. 3a); median tumor specific survival time 12.5 ± 0.9 vs. 17.9 ± 3.0 (Fig. 3b); *p* = 0.012 and *p* = 0.015, respectively). The differences in overall and tumor specific patient survival were still present in male patients of the chemotherapy naive cohort, although losing significance, and for overall survival in women of the chemotherapy naive cohort (Table 1). In the neoadjuvant cohort, MYC score also correlated significantly with overall survival (13.3 ± 1.3 vs. 22.0 ± 1.7 months (Fig. 3c); *p* = 0.018) but not with tumor specific survival (13.1 ± 1.5 vs. 22.4 ± 1.8 (Fig. 3d); *p* = 0.104). Despite these data, finally multivariable survival analysis using the Cox regression showed that the MYC score was not an independent predictor for patient survival neither in the chemotherapy naive nor in the neoadjuvant cohort with FLOT regimen (Supplementary Tables 2, 3).

## Discussion

*MYC* encodes a transcription factor that regulates major processes and signaling pathways, thus playing an indispensable role in cell biology. Consequently, deregulation of *MYC* favors and promotes carcinogenesis. *MYC* amplification is the most common cause of its deregulation in cancer [42]. Here we show that *MYC* amplification is common in GC-GEJ affecting 43.3% (chemotherapy naive cohort) and 63.1% (neoadjuvant cohort) of the patients. It is of tumor biological significance, affected by neoadjuvant chemotherapy and heterogeneous within individual tumors (few scattered single cells up to 100% of the tumor cells). While *MYC* status has been explored previously, our cohort is so far the largest studied, including adenocarcinomas of the GEJ, and the first to consider neoadjuvant treatment schemes (Table 3). Using WMTS instead of tissue microarrays for FISH analysis, we reduced the risk of possible sampling errors and revealed the extent of intratumoral heterogeneity, which might also explain the variability of prevalences of *MYC* amplification in GC reported in previous studies ranging from 7.2% to 72.1% (Table 3) [22–24, 43].

**Table 3:** Findings of previous studies

Study	Country	Cohort size	Method	Therapy naive/ neoadjuvant therapy	Results
Calcagno et al. (40)	Brazil	7	Immunohistochemistry, fluorescence in situ hybridization	Therapy naive	Association of amplification with the intestinal type
Burbano et al. (41)	Brazil	21	Comparative genomic hybridization	Therapy naive	Association of high gene count with the intestinal type
Khaleghian et al. (36)	Iran	102	Chromogenic in situ hybridization	Therapy naive	No Association to clinicopathological patient characteristics
de Souza et al. (37)	Brazil	125	Immunohistochemistry, fluorescence in situ hybridization	Therapy naive	Association of amplification with late onset tumors, the intestinal type and the presence of metastases
Stahl et al. (35)	Germany	109	Fluorescence in situ hybridization	Therapy naive	Association of amplification with the intestinal type and presence of intratumoral heterogeneity
Present study	Germany	592	Fluorescence in situ hybridization	Therapy naive and neoadjuvant therapy	Association of amplification with numerous clinicopathological patient characteristics and patient survival

To our knowledge, we are the first to describe a significant correlation between *MYC* and patient survival. Looking solely at *MYC* amplification in coherent tumor areas we observed no significant survival differences (data not shown). Considering the frequent appearance of single *MYC*-amplified tumor cells we used Cutoff Finder to define a new MYC score. This allowed us to observe that not only the *MYC* amplification in whole tumors or at least in areas of coherent cells, but also single amplified tumor cells influence the tumor biology and the patient outcome. Thus, *MYC* does not need to be homogeneously amplified in the entire tumor to have a tumor biological effect.

Stahl et al. analyzed *MYC* amplification and its heterogeneity in therapy naive GC using tissue microarray technology [24]. *MYC* amplification was found in 24.8%, 85% of these cases showed intratumoral heterogeneity [24]. In our cohort 61.5% of tumors were heterogeneous towards *MYC* amplification. However, we used WMTS and not tissue microarrays, decreasing the risk of a sampling bias. Additionally, our cohort was more extended with 582 patients. De Souza et al. found that increased *MYC* copy numbers are associated with late-onset tumors, an intestinal phenotype and advanced tumor stages, which is concordant with our results. However, the highly significant correlation to the presence of distant metastasis in their study was not seen in our study [22].

We found a highly significant association between *MYC* and the male sex in chemotherapy naive GC-GEJs. Even though previous findings suggest that *MYC* amplification is more prevalent in men and therefore strengthen our results [23, 43], this association has not been found as highly significant as we did. Using binary logistic regression, we additionally were able to prove that sex shows an independent correlation to *MYC*. Li et al. analyzed copy number aberrations on the pan-cancer level and found that the *MYC* gene was a male-dominated copy number gain [44]. This could be a hint that correlation between men and *MYC* could be due to the sex instead of the gene itself further supporting the notion that GC-GEJ biology shows sex specific differences [41].

Interestingly, MYC score 1 tumors showed associations towards a positive HER2-, MET- and p53 status and were more commonly microsatellite stable and EBV-negative. In conjunction with the significant correlation with the non-diffuse histological phenotype according to Lauren (Table 1) this supports the notion that *MYC*-amplified tumors are associated with the molecular subtype of chromosomal instable GCs. These tumors typically also show activation/amplification of receptor tyrosine kinases [25, 26].

To our knowledge, this is the first study comparing the prevalence of *MYC* amplification between chemotherapy naive and neoadjuvantly treated GC-GEJs (Table 1). The number of MYC score 1 tumors was significantly higher in neoadjuvantly treated tumors (63.1% vs. 43.3%) and even correlated significantly with therapeutic response: GC-GEJs with little or no response to neoadjuvant chemotherapy with FLOT were significantly more frequently classified as MYC score 1 reaching 74.6% (Table 1). These data lead to the conjecture that MYC score 1 tumors might undergo some kind of selection during treatment and/or even that *MYC* amplification might predict therapeutic response to neoadjuvant chemotherapy. Further, prospective studies are urgently needed to test this hypothesis as this might have therapeutic implications.

### Limitations

Our retrospective observational study does not provide mechanistic insight into the tumor biological effects of *MYC* in GC-GEJ. We also did not perform in-depth molecular biological analyses that might further elucidate the genetic context in which *MYC* amplification occurs. However, we intended to investigate the prevalence and thus the putative tumor biological relevance of *MYC* amplification in GC-GEJ using whole-mount tissue sections and fluorescence in situ hybridization. The MYC score was also correlated with clinicopathologic patient characteristics that have not been the focus of previous studies, such as a positive correlation with HER2, MET, p53 and SARIFA and a negative correlation with MSI and EBV, providing novel data.

## Conclusion

Summing up, our study describes the analysis of *MYC* amplification in the so far largest Caucasian GC-GEJ cohort. We show that *MYC* amplification is highly prevalent, shows intratumoral heterogeneity, is sex dependent and correlates negatively with tumor regression following neoadjuvant chemotherapy with the FLOT regimen. Prospective studies need to proof and validate independently whether *MYC* amplification could predict therapeutic response to neoadjuvant chemotherapy.

## Supplementary Information

Supplementary Material 1: Suppl. Table 1. Correlation of MYC score with clinicopathological patient characteristics, separately for proximal and distal tumors.

Supplementary Material 2: Suppl. Table 2 Multivariable survival analysis of the chemotherapy naive cohort. All p-values < 0.100 in the univariate survival analysis were included in the multivariable survival analysis. Results are shown separately for overall and tumor specific survival. In the chemotherapy naive cohort pN category, presence of metastases, lymph node ratio, perineural invasion, resection status, MET status and SARIFA showed to be independent prognostic factors of overall survival and pN category, presence of metastases, perineural invasion, resection status, MET status and SARIFA of tumor specific survival.

Supplementary Material 3: Suppl. Table 3 Multivariable survival analysis of the neoadjuvant cohort. All p-values < 0.100 in the univariate survival analysis were included in the multivariable survival analysis. Results are shown separately for overall and tumor specific survival. In the neoadjuvant cohort presence of metastases, venous invasion and resection status were found to be independent prognostic factors of overall survival and venous invasion and resection status for tumor specific survival.

## Data Availability

The datasets generated and/or analyzed during this study are available from thecorresponding author upon reasonable request.

## Abbreviations

GC: Stomach Cancer
GEJ: Gastroesophageal Junction
EBV: Epstein-Barr Virus
FLOT: Fluorouracil, Leucovorin, Oxaliplatin, Docetaxel
PD-1: Programmed cell Death protein 1
HER2: Human Epidermal Growth Factor Receptor 2
TRG: Tumor Regression Grade
WMTS: Whole Mount Tissue Section
FISH: Fluorescence in Situ Hybridization
UICC: Union Internationale Contre le Cancer
H. pylori: Helicobacter Pylori
Histoscore: HScore
MSI: Microsatellite-instability
SARIFA: Stroma AReactive Invasion Front Area
ITBCC: International Tumor Budding Consensus Conference
dH_2_O: Deionized Water
SSC: Standard Saline Citrate
DAPI: 4´,6-diamidino-2-phenilindole
CISH: Chromogenic in Situ Hybridization
